# Tension Pneumothorax Requiring Video‐Assisted Thoracoscopic Surgery With Multimodal Pleurodesis: An Unexpected Ramification of a Ground Level Fall Without Significant Blunt or Penetrating Injury

**DOI:** 10.1002/rcr2.70132

**Published:** 2025-02-27

**Authors:** Christ Ordookhanian, Max Slosarski, Ryan F. Amidon, Komal Kapoor

**Affiliations:** ^1^ School of Medicine, Department of Internal Medicine University of California Riverside California USA; ^2^ School of Medicine Idaho College of Osteopathic Medicine Meridian Idaho USA; ^3^ School of Medicine Medical College of Wisconsin Milwaukee Wisconsin USA

**Keywords:** cardiothoracic surgery, ground‐level fall, pleurodesis, tension pneumothorax, video‐assisted thoracoscopic surgery (VATS)

## Abstract

Tension pneumothorax is characterised by progressive air accumulation in the pleural space leading to increasing intrathoracic pressure and haemodynamic instability. Pneumothorax is generally observed following blunt or penetrating trauma, though spontaneous pneumothorax can also occur in patients with risk factors. This case highlights the potential for severe complications in an otherwise healthy patient with tension pneumothorax resulting from a ground‐level fall onto an absorbent soft surface with mild indirect thoracic trauma. Initial respiratory status coupled with asthma history was worrisome for asthma exacerbation; however, imaging confirmed tension pneumothorax, later complicated by persistent air leak associated with apical blebs, corrected surgically.

## Graphical abstract

This case highlights the potential for severe complications in an otherwise healthy patient with tension pneumothorax resulting from a ground‐level fall onto an absorbent soft surface with mild indirect thoracic trauma. Initial respiratory status, coupled with asthma history, was worrisome for asthma exacerbation; however, imaging confirmed tension pneumothorax, later complicated by a persistent air leak associated with apical blebs, corrected surgically.

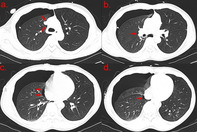

## 1 Introduction

Pneumothorax, or the presence of air in the pleural space, can be divided into several categories. Tension pneumothorax involves progressive accumulation of air leading to increased intrathoracic pressure, compressing nearby structures and potentially causing haemodynamic instability. Spontaneous pneumothorax can arise without major trauma and is categorised as primary spontaneous, typically occurring in young, healthy individuals, and secondary spontaneous, which occurs in patients with underlying lung pathology. Traumatic pneumothorax occurs following penetrating trauma, allowing external air to enter the pleural space and accumulate [1].

The clinical presentation of pneumothorax is variable, often including chest pain, shortness of breath, tachycardia, hypoxia, decreased breath sounds, and hyperresonance to percussion on the affected side. Pneumothorax may spontaneously result from minimal trauma or physical exertion, especially in individuals with lung fragility. Chest x‐rays (CXR) remain the most common diagnostic modality for suspected traumatic pneumothorax, though lung ultrasound is emerging as a promising alternative; computed tomography (CT) is the gold standard, though is rarely utilised for initial evaluation.

Small, asymptomatic pneumothoraces are managed conservatively, while larger or symptomatic cases require interventions ranging from chest tube placement to thoracoscopy [1]. Tension pneumothorax uniquely requires urgent needle decompression followed by chest tube placement. While large‐bore chest tubes have been traditionally preferred, small‐bore pigtail catheters are increasingly utilised, largely due to comparable efficacy with reduced procedural pain [2]. Chest tubes are connected to an external single‐direction valve system attached to a vacuum with a water seal that allows air to exit from the pleural space on exhalation and prevents air from entering during inspiration. The chest tube is left in place until no air bubbles are visualised escaping into the water seal and a repeat CXR confirms pneumothorax resolution [3]. If persistent after 3–5 days, which often occurs in the setting of a bronchopleural fistula, surgery may be required as the prolonged presence of a chest tube with persistent air leak may contribute to iatrogenic events, with the most severe being infection.

## 2 Case Report

A middle‐aged male with a history of intermittent asthma and tobacco use (50‐pack‐year history) not on any medications presented to the emergency department with chest pain. He reported tripping, striking his torso on the grass, before walking away without any symptoms. Approximately 30 min post‐fall, he experienced severe, rapid‐onset chest pain with shortness of breath. Emergency medical services found him hypertensive at 191/111 mmHg and tachycardic at 137 beats per minute, saturating at 96% on room air.

**FIGURE 1:**
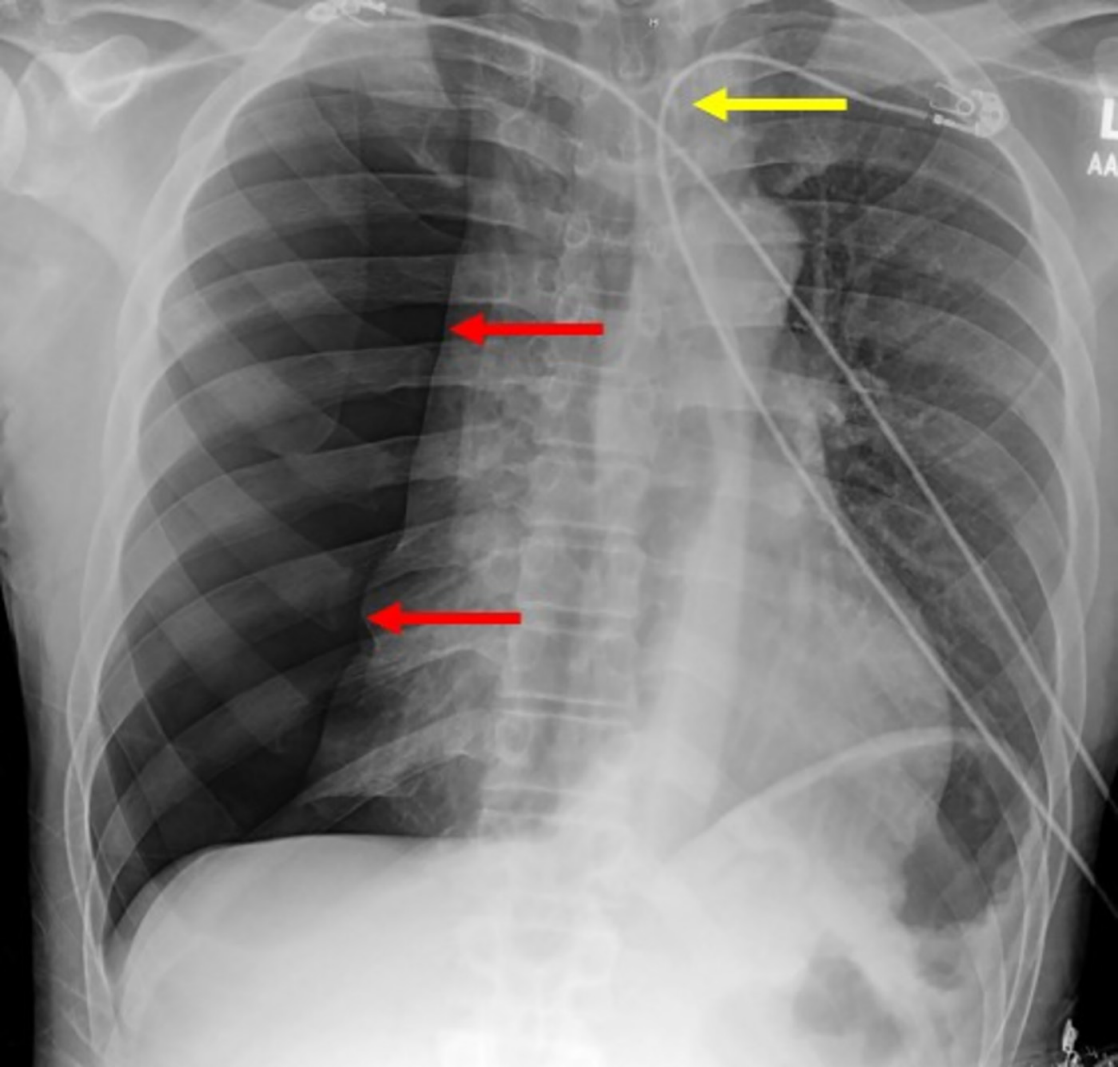
Initial chest x‐ray revealing right‐sided pneumothorax (red arrows) with leftward tracheal deviation (yellow arrow), characteristic of tension pneumothorax.

In the emergency department, his blood pressure normalised with reduced tachycardia. Complete metabolic panel, complete blood count, and arterial blood gas were unremarkable. Urine drug screen was positive for cannabinoids. An electrocardiogram demonstrated sinus tachycardia, and troponin levels were within the reference range. The care team suspected asthma exacerbation, and the patient promptly received albuterol with a metered dose inhaler and prednisone 40 mg. However, a CXR revealed a right‐sided pneumothorax with complete collapse of the right lung and left‐sided midline shift, consistent with tension pneumothorax (Figure 1).

**FIGURE 2:**
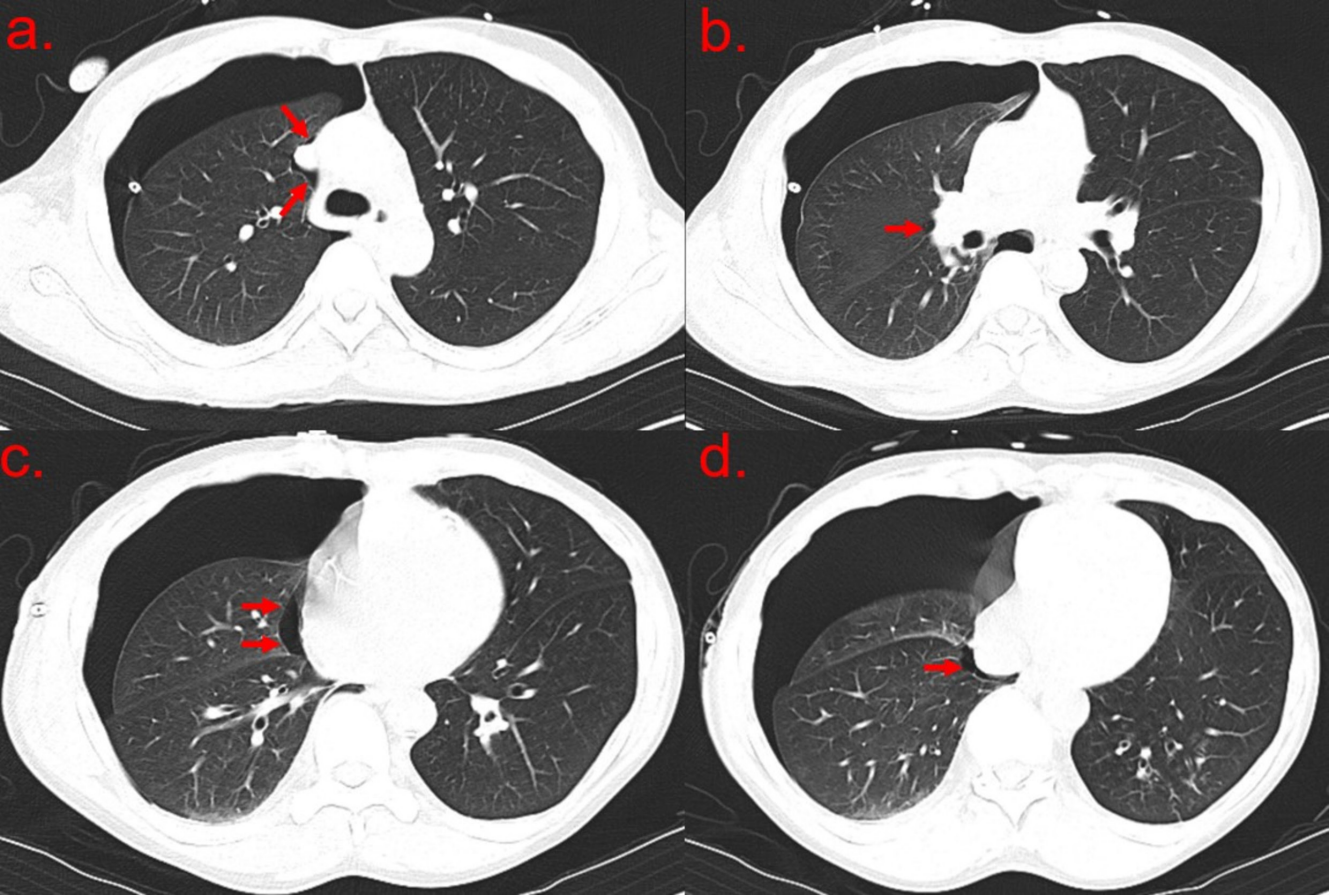
Pre‐operative CT revealing blebs and bullae (red arrows).

**FIGURE 3:**
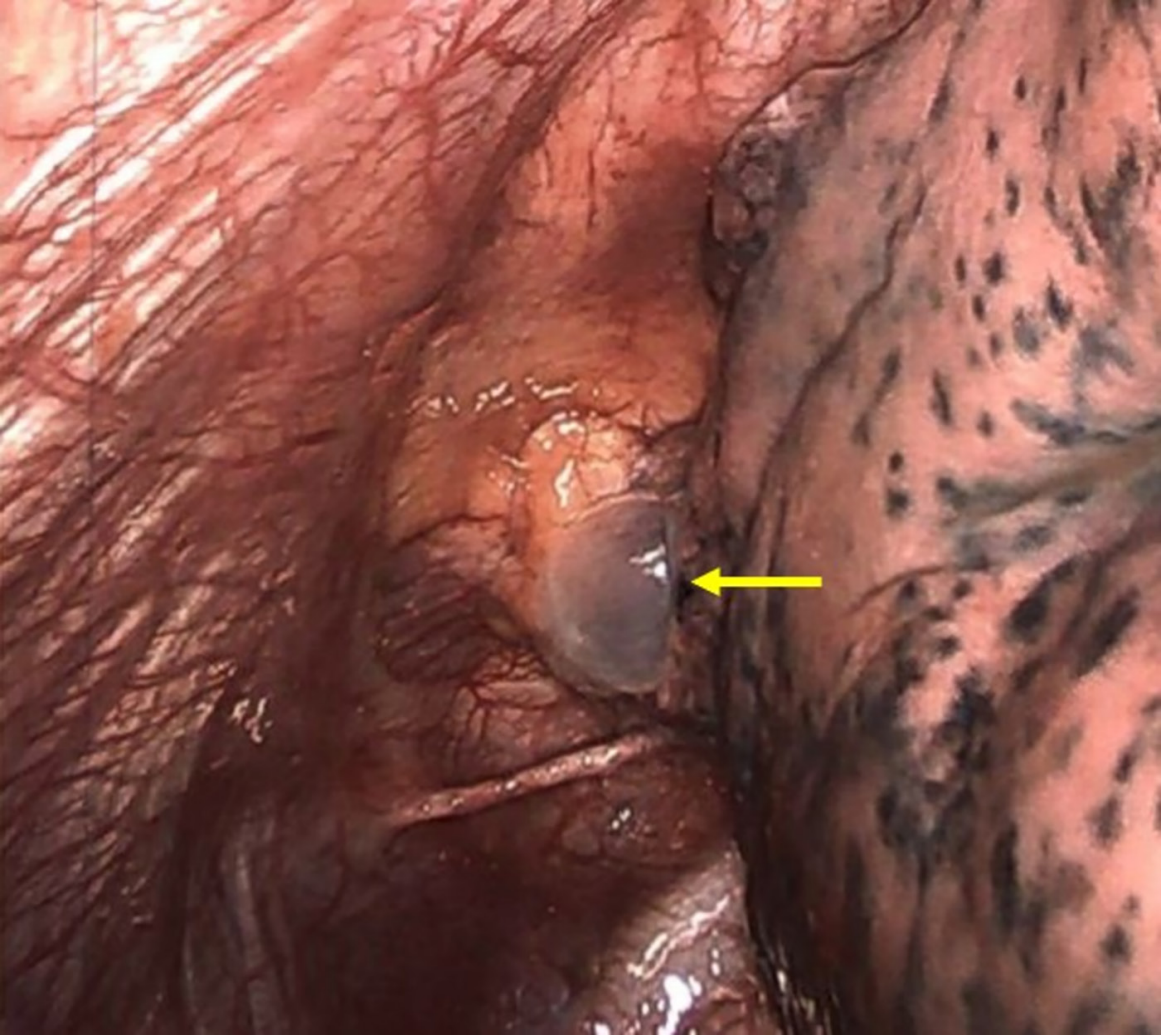
Intra‐operative apical bleb (yellow arrow), corresponding to that observed on computed tomography imaging.

A right small‐bore pigtail pleural catheter was placed, resolving most of the pneumothorax, and the patient was admitted. Unfortunately, the pneumothorax was non‐resolving with constant chest tube suction. Bubbles were always present on the chest drainage system. Cardiothoracic surgery was consulted on day 3 of admission, recommending a preoperative pulmonary function test with spirometry (Table 1) and arterial blood gas, which were unremarkable. Computed tomography (CT) revealed apical blebs suspicious for persistent bronchopleural fistula with air leak (Figure 2). Video‐assisted thoracoscopic surgery (VATS) with apical blebectomy and pleurodesis (mechanical to the apical and lateral chest wall and chemical with doxycycline) was performed (Figure 3), resulting in symptomatic improvement. Chest tubes were kept to water seal for 2 days before removal on discharge. The CXR on the day of discharge revealed only a trace apical pneumothorax. On two subsequent outpatient follow‐up visits, the patient reported feeling back at baseline without concerns.

**TABLE 1:** Pulmonary function test (spirometry) with unremarkable results.

	Pred^a^	Pre^b^	% (Pre/Pred)^c^	Post^d^	% (Post/Pred)^e^	%Chg (Post/Pre)^f^
VC MAX^g^	5.09	3.79	75	4.09	80	8
FVC^h^	3.95	3.72	94	4.09	103	10
FEV1^i^	3.13	3.26	104	3.27	105	0
FEV1/FVC^j^	79	86	109	80	101	−7
MVV^k^	127	82	65	N/A^o^	N/A^o^	N/A^o^
PEF^l^	8.42	7.42	88	5.30	63	−29
FEF25‐75%^m^	2.83	4.12	146	3.45	122	−16
FEV3^n^	4.12	3.68	89	3.82	93	4

*Note:* While forced expiratory volume in 1 s over forced vital capacity (FEV1/FVC) was high pre‐treatment, a sign of no significant obstructive disease, its subtle decrease post‐treatment may indicate mild restrictive disease. Peak expiratory flow (PEF) and forced expiratory flow at 25%–75% of forced vital capacity (FEF25‐75%) decreasing post‐treatment are also consistent with restrictive lung disease. Importantly, pneumothorax can lead to restrictive lung patterns, making these results expected. Additionally, restrictive features are also associated with large bullae that restrict lung volume.

^a^ Predicted value.

^b^ Pre‐bronchodilator measure.

^c^ Percent of pre‐bronchodilator value over predicted value.

^d^ Post‐bronchodilator measure.

^e^ Percent of post‐bronchodilator value over predicted value.

^f^ Percent change from pre‐bronchodilator value to post‐bronchodilator value.

^g^ Maximum vital capacity.

^h^ Forced vital capacity.

^i^ Forced expiratory volume in 1 s.

^j^ Forced expiratory volume in 1 s over forced vital capacity.

^k^ Maximum voluntary ventilation.

^l^ Peak expiratory flow.

^m^ Forced expiratory flow at 25%–75% of forced vital capacity.

^n^ Forced expiratory volume in 3 s.

^o^ Not applicable.

## 3 Discussion

Blebs, or bullae, frequently contribute to persistent air leaks and are associated with asthma, chronic obstructive pulmonary disease, and smoking (tobacco and cannabis). The formation of these blebs results from local emphysema from small airway injury induced by tobacco and cannabis [4, 5]. Additionally, asthma is associated with bleb rupture from increased pleural pressures triggered by bronchospasm and hyperinflation [6]. The lung apex is the most common location for bleb formation and this position increases the likelihood of bleb rupture and subsequent pneumothorax due to greater negative pressure at the lung apex [7]. If the air leak from a ruptured bleb communicates directly with a bronchus, a bronchopleural fistula may be created, complicating pneumothorax resolution [8].

Surgical options include VATS and traditional open thoracotomy to identify the source of air leak(s), clamp pulmonary blebs, and complete a pleurectomy (removal of pleural lining for the lung to adhere directly to the chest wall) or pleurodesis (insertion of irritating compounds such as doxycycline into pleural space, inducing an inflammatory reaction for the lung to adhere to the chest wall) [9, 10]. VATS is preferred as it is associated with a reduction in admission length, postoperative complications, pain, and risk of infection [3]. While surgery poses risks such as infection, respiratory distress, and pleural effusion, it often provides a definitive solution to persistent or recurrent pneumothoraces.

Importantly, providing treatment in a timely manner requires prompt diagnosis. In our case, the patient's history of asthma, respiratory distress on presentation, and lack of significant trauma led to the assumption that this was most likely an asthma exacerbation. It was not until the CXR revealed a pneumothorax that this diagnosis was considered, delaying treatment. Other signs that could have elevated pneumothorax on the differential include the sudden‐onset chest pain and asymmetric breath sounds on auscultation (decreased on affected side), in addition to the lack of characteristic chest tightness and wheezing experienced with asthma exacerbation.

This case highlights important considerations in persistent pneumothorax management. Patients with risk factors such as smoking, pre‐existing lung conditions, or anatomical defects (i.e., apical blebs) are more likely to require surgery. Timely surgical referral can prevent life‐threatening complications such as worsening pneumothorax, mediastinal shift, and tension pneumothorax. Close monitoring of pleural drainage with repeat imaging allows the care team to determine when the transition to surgical management is appropriate. By addressing our patient's underlying anatomical issues, pneumothorax resolution was achieved.

## Author Contributions

All authors have reviewed the final version to be published and agreed to be accountable for all aspects of the work. Christ Ordookhanian's contributions included acquisition, analysis, and interpretation of data, manuscript review, and concept and design. Max Slosarski's contributions included analysis and interpretation of data, drafting of the manuscript, and manuscript review. Ryan F. Amidon's contributions included analysis and interpretation of data, drafting of the manuscript, and manuscript review. Komal Kapoor's contributions included analysis and interpretation of data, manuscript review, and concept and design.

## Ethics Statement

The authors declare that appropriate written informed consent was obtained for the publication of this manuscript and accompanying images.

## Conflicts of Interest

The authors declare no conflicts of interest.

## Data Availability

Data sharing not applicable to this article as no datasets were generated or analyzed during the current study.
